# What is health and what do we mean when we say an intervention improves health?

**DOI:** 10.1038/s41431-023-01388-8

**Published:** 2023-06-21

**Authors:** Fiona Ulph

**Affiliations:** Manchester Centre for Health Psychology, Division of Psychology & Mental Health, School of Health Sciences, Faculty of Biology, Medicine and Health, University of Manchester, Manchester Academic Health Science Centre, Oxford Road, Manchester, M13 9PL UK

**Keywords:** Psychology, Health care

Newborn bloodspot screening (NBS) programmes are seen as one of the greatest advances in public health and undoubtedly do improve the physical health outcomes of many in a way that should be highly valued. A common narrative about the benefits of NBS is that it removes the ‘diagnostic odyssey’ for families and this is being amplified in the current debates about using whole genome sequencing in this screening programme. In this issue of the *European Society of Human Genetics* Raspa et al. [1] explored the experience of parents who receive a diagnosis of SCID via newborn screening. A key message from this work is that, in line with the World Health Organisation’s definition of health, it is important to view health more broadly as the physical, mental and social well-being of a person. Their paper highlights that although identifying babies with SCID appears to have significant physical benefits, parents can experience a range of psychological reactions, which warrant more careful consideration about how we prepare and support parents to receive a diagnosis via NBS.

Although previous research has shown that parents can experience adjustment challenges to a range of NBS results [2], this work is novel as it maps parents’ experiences across time. Through this, it is possible to see that although we might remove the diagnostic odyssey for parents via NBS, by providing valuable diagnoses, these parents continue with feelings of uncertainty, weight of responsibility, and concern.

Through their analysis, they also separated the emotional and cognitive elements of parents’ responses which fits with models of illness understanding used in Health Psychology [3]. This helps to show the complexity of parents’ reactions to such diagnoses. This separation also has the benefit of highlighting that although the experiences in the accounts could be seen as understandable adjustments to a serious diagnosis, there are elements where we could intervene and reduce distress.

The parents in the Raspa et al. study all spoke English and were part of support groups. Whilst this could be seen as a limitation, looking at the stressors experienced, it arguably strengthens their argument for concern as parents outside of support groups may find such experiences even more challenging. Although understanding parents’ reactions to SCID diagnoses is a nascent field, this work fits in a larger context illustrating how NBS results are challenging our existing personal medical models [4], and currently, parents receiving results can struggle to find a place in existing support networks [5]. Their work adds to the call to that to truly unlock the potential of optimising health via NBS, we must consider the mental and social support needs on a par with the physical [2].
